# Engineered plastic-associated bacteria for biodegradation and bioremediation

**DOI:** 10.1186/s44314-024-00007-0

**Published:** 2024-07-15

**Authors:** Arianna Schneier, Gavin Melaugh, Joanna C. Sadler

**Affiliations:** 1 Institute of Quantitative Biology, Biochemistry and Biotechnology, School of Biological Sciences, University of Edinburgh, Roger Land Building, Alexander Crum Brown Road, King’s Buildings, Edinburgh, EH9 3FF UK; 2 School of Physics and Astronomy, University of Edinburgh, Edinburgh, EH9 3FD UK; 3 School of Engineering, University of Edinburgh, Edinburgh, EH9 3JL UK

**Keywords:** Plastic degradation, Plastic adhesion, Biofilms, Biotechnology, Microplastics

## Abstract

The global plastic waste crisis has triggered the development of novel methods for removal of recalcitrant polymers from the environment. Biotechnological approaches have received particular attention due to their potential for enabling sustainable, low-intensity bioprocesses which could also be interfaced with microbial upcycling pathways to support the emerging circular bioeconomy. However, low biodegradation efficiency of solid plastic materials remains a bottleneck, especially at mesophilic conditions required for one-pot degradation and upcycling. A promising strategy used in nature to address this is localisation of plastic-degrading microbes to the plastic surface via biofilm-mediated surface association. This review highlights progress and opportunities in leveraging these naturally occurring mechanisms of biofilm formation and other cell-surface adhesion biotechnologies to co-localise engineered cells to plastic surfaces. We further discuss examples of combining these approaches with extracellular expression of plastic-degrading enzymes to accelerate plastic degradation. Additionally, we review this topic in the context of nano- and microplastics bioremediation and their removal from wastewater and finally propose future research directions for this nascent field.

## Introduction

Biocatalysis holds vast potential to address the plastic waste crisis [1–4] through depolymerisation of traditionally recalcitrant materials under low process-intensity conditions [5–7]. A surge in research focusing on plastic biodegradation over the past decade is providing an expanding toolbox of biocatalysts for the degradation of many synthetic polymers, in particular hydrolysable plastics such as poly(ethylene terephthalate) (PET) [8–12], poly(lactic acid) (PLA) [13–15] and poly(urethanes) [16, 17]. Additionally, the emerging field of plastic bio-upcycling has demonstrated that plastic degradation products can serve as feedstocks for the production of value-added, second-generation chemical products which could enable a more sustainable and circular plastics economy [18–26].

A challenge inherent to plastic degradation is the heterogeneity of reaction mixtures. Unlike homogenous biocatalytic processes in which the substrate and enzyme are both solvated in the aqueous phase, the enzymatic degradation of plastics is heterogeneous under mesophilic conditions. Thus enzyme-plastic reactions are constrained to the interface between the solid plastic surface and the liquid phase. Therefore, two important strategies to improve plastic biodegradation efficiency are to localise the biocatalyst to the phase interface and to increase the surface area of the plastic substrate. Indeed, one of the most promising enzyme candidates for plastic degradation from nature, PETase from *Ideonella sakaiensis (IsPETase)* [10], has a flat hydrophobic surface surrounding the substrate binding cleft. This has been proposed to aid the co-localisation of the enzyme to the PET surface and is thought to contribute to the remarkable efficiency of this enzyme [27, 28].

At the cellular level, microorganisms address this challenge in nature by colonising solid, abiotic surfaces as biofilms. A biofilm is a complex and dynamic three-dimensional assemblage of microorganisms encapsulated within an extracellular polymeric matrix associated with a solid surface. Biofilm formation is controlled by a vastly complex regulatory network in response to environmental stressors, including reactive oxygen species, antibiotics, heat and pH fluctuations [29–31]. Encapsulation within the biofilm matrix provides microbial populations with enhanced stability towards these high-stress conditions. As such, biofilms are frequently associated with infection, pathogenicity and contamination in biomedical settings and have been the focus of intensive study with regards to their prevention and removal. However, these properties also make controlled or engineered, non-pathogenic biofilms an attractive tool for biotechnological applications, perhaps most notably in wastewater treatment and bioremediation [32–34]. Biofilms have also been exploited to improve biocatalyst performance via the expression of enzymes in biofilm-associated cells. Enzymes expressed in these systems have have demonstrated increased longevity and stability compared to purified enzymes, which has been attributed to rapid enzyme regeneration [35, 36].

Whilst mechanisms and regulation of biofilm formation vary across species, and even between different strains within the same species, the universal first step of biofilm formation is adhesion [37–40]. Depending upon the context of the biofilm, this may be adhesion to other cells or abiotic surfaces such as plastics. In the latter stages of biofilm development, a protective extracellular polymeric matrix is formed, comprising oligosaccharides, DNA and proteins, collectively known as extracellular polymeric substances (EPS). The study of these biofilm-formation mechanisms of adhesion and EPS production has provided a useful starting point for the rational control and engineering of microbial biofilms for biotechnological application. In particular, research efforts have focused on the use of surface-anchored filamentous proteins such as curli [41], antigen-43 (Ag43), fimbriae and pili, and EPS [38] to control biofilm formation on a range of abiotic surfaces.

**Fig. 1:**
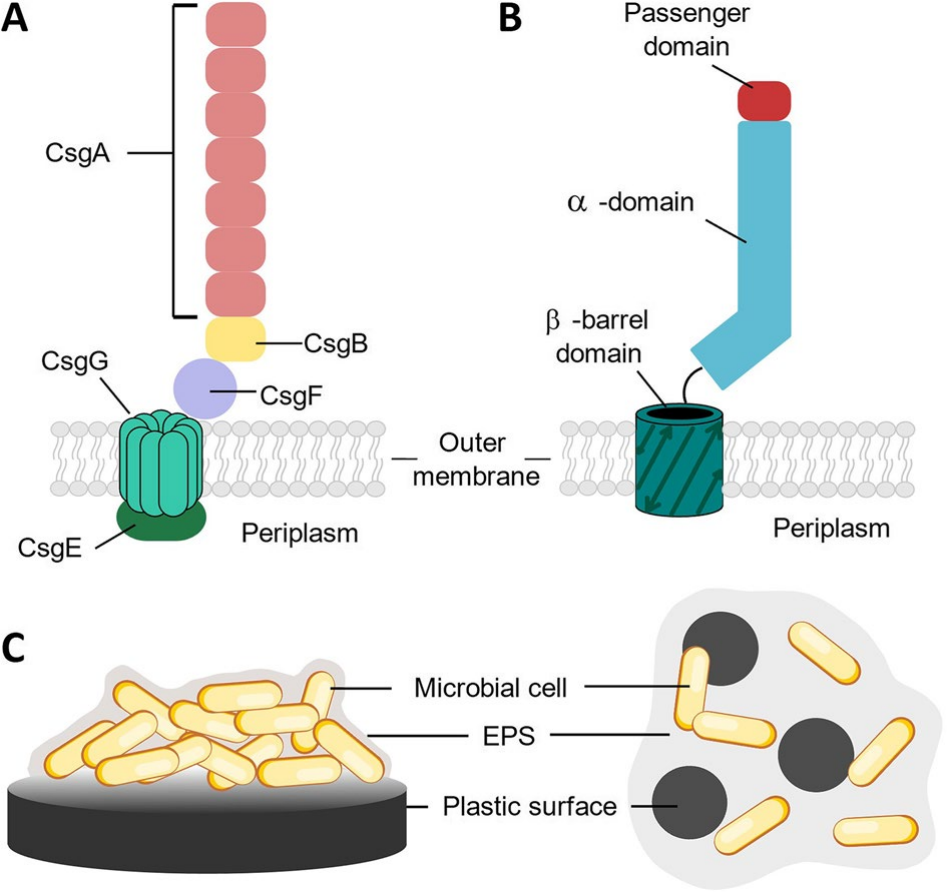
Examples of natural mechanisms of bacterial association with plastic surfaces in microbial biofilms which have been leveraged for addressing adhesion, encapsulation or degradation of plastic. **A** Curli fibre. **B** Antigen-43 (Ag43). **C** Extracellular polymeric substances (EPS) surrounding microbial cells associated with a macroscopic plastic surface (left) or microplastics (right)

In this review, we discuss examples of this in the context of plastic biodegradation and bioremediation. In particular, bioengineering strategies to control microbial adhesion to and interaction with hydrophobic plastic surfaces using Ag43, curli and EPS (Fig. 1) are explored. We also discuss examples of biotechnologies which leverage other naturally occurring methods of adhesion to hydrophobic surfaces, such as hydrophobins and adhesins. Naturally occurring biofilms on plastic surfaces and laboratory propagation thereof are discussed in depth elsewhere [42–45] and are therefore out of scope of this review. We first review engineering strategies for enhanced bacterial adhesion to bulk plastic, followed by a discussion of progress in the field of engineering surface-associated bacteria for plastic depolymerisation through extracellular expression of degradative enzymes. We next review progress in the field of engineering microbial systems for the encapsulation and bioremediation of microplastics and finally propose future directions and opportunities in this emerging field. Whilst other recent reviews include examples of bacterial adhesion to plastic surfaces using engineered bacteria [2, 46, 47] and biocatalyst engineering to affinity for plastic surfaces [11], to the best of our knowledge this if the first review to focus solely on bioengineering strategies for co-localisation of microorganisms and plastic surfaces, discussed in the context of both bulk plastics and microplastics.

## Engineered microbes for enhanced plastic adhesion

Characterisation of bacterial adhesion mechanisms has prompted further study into the rational design and optimisation of methods to anchor microbial cells to plastic surfaces examples of which are summarised in Table 1. For example, Leech and co-workers demonstrated a ~ 7-fold increase in biomass adhesion to poly(tetrafluoroethylene) (PTFE), compared to the control strain BL21, by PHL644. PHL644 is a K-12 derivative strain of *Escherichia coli* which overexpresses the positive regulator of the curli operon, CsgD, via inclusion of the *ompR234* allele. This results in high levels of curli production under standard cultivation conditions [48]. This resulted in increased biofilm formation and therefore increased cellular adhesion ability. Biofilm formation on plastic surfaces was also found to loosely correlate with surface hydrophobicity, which is known to be one of the key surface property determinants of biofilm formation [48, 49].

**Table 1:** Summary of engineering strategies for enhanced microbial adhesion to plastic surfaces

Plastic	Host	Genetic modification	Effect on plastic adhesion	Reference
PTFE	*E. coli* PHL644	*ompR234* allele inclusion	~ 7-fold increase compared to *E. coli* BL21	Leech et al. [48]
PS	*E. coli* MG1655	Ag43 overexpression (arabinose inducible)	~ 4.8-fold increase compared to wild-type in dark	Jin and Riedel-Kruse [50]
		Ag43 overexpression (light inducible)	~ 2.7-fold increase in illumination compared to dark	
PS	*E. coli* MG1655 MGJ1	OxyR^red^ overexpression	~ 2-fold increase compared to wild-type	Schembri et al. [51]
PDMS	*E. coli* XL10-Gold	DOPA-histidine fused to OmpW	~ 15,000 cells/mm^2^	Park et al. [52]
PET				
PU			~ 6500 cells/mm^2^	

Antigen-43 (Ag43) has also been leveraged as a tool for adhering microbial cells to plastic surfaces. For example, overexpression of the *E. coli* K-12 *flu* gene, which encodes the multi-domain Ag43 protein (Fig. 1B), using a light-inducible promoter enabled high-resolution cell patterning on polystyrene surfaces [50]. A positive correlation between illumination intensity and biomass adhesion was observed, and biomass adhesion also increased linearly with illumination time up to 8 h. This tightly controlled system enabled generation of a ‘biofilm lithography’ system, whereby microbial adhesion to the surface could be controlled by application of a photomask. Ag43 expression, and therefore bacterial adhesion to abiotic surfaces, can also be modulated via engineering the redox state of the cellular redox sensor OxyR. Schembri and co-workers used mutants of OxyR locked into either the reduced (OxyR^red^) or oxidised (OxyR^ox^) state to modulate *flu* gene expression. Overexpression of OxyR^ox^ resulted in a ~ 2-fold increase in biomass adhesion to a polystyrene surface in comparison to OxyR^WT^ and OxyR^red^ [51]. Whilst these data were obtained as part of a wider study into the factors regulating *flu* gene expression, it demonstrates the potential for targeting the biofilm regulatory network to modulate bacterial adhesion to plastic surfaces.

A further example of engineering *E. coli* to adhere to solid surfaces was inspired by the mechanism of attachment of mussels to inorganic and organic surfaces in nature. This is modulated by a polypeptide comprising repeating units of 3,4-dihydroxyl-L-phenylalanine (DOPA) and lysine, and DOPA and histidine [53]. In this study, the authors used the outer membrane protein W (OmpW) as the cell membrane anchor in combination with the perhydrolase from *Pseudomonas aeruginosa* (PerPA) as a functional spacer protein, from which they displayed a mussel protein-inspired catecholamine cargo. A tyrosinase was then used to convert the Tyr side chain to DOPA via *meta*-hydroxylation [52]. The resultant cells showed adherence to all abiotic surfaces tested (Au, Si, Ti, PET, poly(urethane) (PU) and poly (dimethylsiloxane) (PDMS)). Whilst PET and PDMS showed a similar level of adhesion (~ 15,000 cells/mm^2^), cells had poorer adhesion to PU (~ 6500 cells/mm^2^). Adhesion to PET and PU was hypothesised to be due to *π*-*π* stacking interactions between the polymer chain and catechol moiety, whereas adhesion to PDMS was theorised to be due to electrostatic interactions between surface oxidised PDMS and the amine group of catecholamine. By further engineering of the cells to co-display a degradative enzyme, this nature-inspired approach to cellular adhesion could be a useful method to co-localise functional biomass and plastic surfaces. It is noteworthy that the plastic surface itself may also be modified to promote biofilm formation, although this is out of scope of the present review [49, 54].

## Engineered microbes for bulk plastic adhesion and degradation

Methods to adhere biomass to abiotic surfaces enable researchers to study fundamental interactions of microbes with plastic surfaces. It also offers an ideal platform for the presentation of functional biomass to the plastic surface to perform a useful function, such as plastic depolymerisation and degradation. Examples of these technologies are summarised in Table 2 and described in detail below.

**Table 2:** Summary of engineered microorganisms for plastic degradation enhanced by surface adhesion

Plastic	Host	Biocatalyst	Display system	Substrate	Degradation assay conditions	Degradation efficiency (productivity)	Reference
PET	*E. coli* PHL628	*ls*PETase	N-terminal fusion to CsgA	Commercial PET; 37% crystallinity	7 days, 30 °C	4.79%^a^ (0.29–0.33 g/L TA)	Zhu *et al.* [55]
				Wastewater bottle microplastics; 28% crystallinity	7 days, 30 °C	7.43%^a^	
				Wastewater bottle microplastics; 28% crystallinity	7 days, 30 °C, 0.02% STS	9.10%^a^	
PET	*E. coli* BL21(DE3)	*ls*PETase	N-terminal fusion to FadL; C-terminal fusion to hydrophobin *Tr*HFBII	Wastewater bottle films (crystallinity not reported)	7 days, 37 °C (media supplemented on day 4)	*Not reported* (0.30–0.35 g/L TA)	Jia *et al.* [56]
PET	*Pichia pastoris*	*ls*PETase	N-terminal fusion to GCW51; Co-display of *Tr*HFBI via N-terminal fusion to GCW61	Commercial film; 45% crystallinity	18 h, 30 °C	3.20%^b^	Chen* et al. *[57]
				Commercial film; 6% crystallinity	18 h, 30 °C	55.00%^b^	
PET	*E. coli* BL21(DE3)	FAST-PETase	N-terminal fusion to YfaL	Amorphous PET from	24 h, 30 °C	6.96%^b^	Hu and Chen [58]
			Co-display with INP-cp52k	Commercial PET bottles		9.47%^b^	
			Co-display with INPNC-mfp-3			15.73%^b^	
PCL	*E. coli* BL21(DE3)	Dh3	*n/a (biocatalysts secreted)*	1% w/v PCL beads	5 days, 37 °C	< 40%^c^	Howard and McCarthy [59]
		Dh3 and DgcC				40–60%^c^	
		Dh3 and WspR				40–60%^c^	

*STS* sodium tetradecyl sulfate, *TA* terephthalic acid, *INPNC* truncated form of ice nucleation protein containing the N- and C-terminal portions

^a^Degradation rate based on TA and MHET production

^b^Degradation rate measuring TA, MHET and BHET production over time

^c^Weight loss using an inoculated media control with PCL beads

Research in this field to date has largely focused on degradation of PET, in part due to a rapidly expanding toolbox of PET depolymerases which operate at mesophilic conditions close to 30 °C [8, 10, 60–63]. These biocatalysts have been successfully produced using a range of expression technologies, including cell-free protein expression [64], secretion of free enzyme into the extracellular environment [65, 66] and surface display [67, 68]. Out of these, PETase cell-surface display technologies have been successfully paired with cellular adhesion to PET surfaces to expediate depolymerisation.

**Fig. 2:**
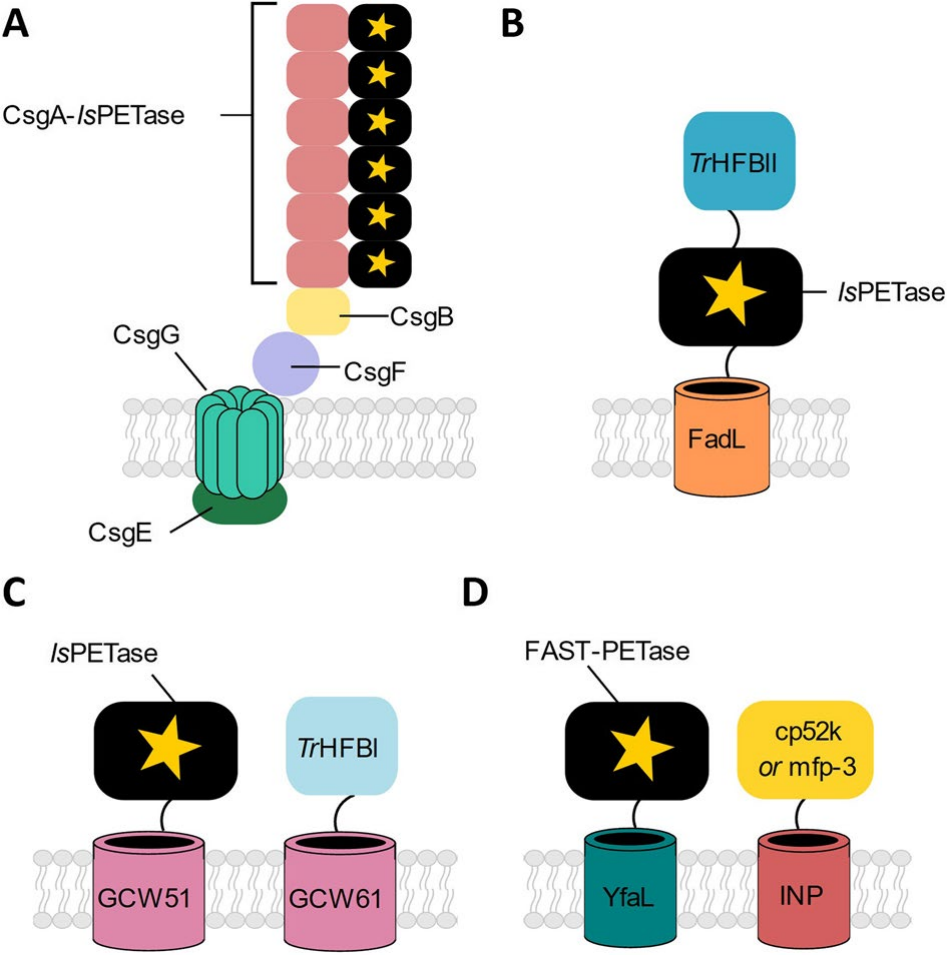
Examples of surface display of PET-degrading biocatalysts, *ls*PETase and FAST-PETase, using **A** engineered curli fibres, **B** and **C** co-display of hydrophobins or **D** co-display of adhesin proteins. CsgA-F, subunits of the curli fibre assembly; FadL, an *E. coli* transmembrane protein; *Tr*HFBI/II, class I/II type hydrophobin from Trichoderma reesei; GCW51/61, anchoring proteins from *Pichia pastoris*; YfaL, AIDA-I family autotransporter from *E. coli*; cp52k and mfp-3, hydrophobic adhesins from mussels; INP, ice-nucleation protein

For example, engineered curli fibres were shown to be an effective tool to improve the efficiency of PET degradation. Zhu *et al*. genetically fused *ls*PETase to the CsgA subunit of curli fibres in *E. coli* PHL628 to generate a whole-cell biocatalyst in a process they term ‘biofilm-integrated nanofiber display (BIND) strategy’ [69], in this instance generating BIND-PETase [55] (Fig. 2A). The enzymatic activity of BIND-PETase was demonstrated via hydrolysis of *p*-nitrophenyl butyrate, which generates a highly fluorescent product upon hydrolysis. BIND-PETase degraded 4.8% of semicrystalline PET after 7 days at 30 °C, whilst purified, cell-free PETase lost activity after 24 h. BIND-PETase was also found to degrade microplastics from water treatment plants, water bottles and egg trays [55]. This work demonstrates potential of surface-adhered engineered microbes to improve plastic degradation efficiency through improved stability and longevity, and lower biocatalyst production costs.

The surface display of plastic-degrading enzymes has also been paired with additional heterologous proteins to improve the surface adhesion of the microbe to the plastic substrate. A key example of this is co-expression of fungal hydrophobins in the engineered host. Fungal hydrophobins are secreted amphiphilic proteins [70]. The fusion of free PET-degrading enzymes to hydrophobins or their co-expression as a second surface-displayed protein has been shown to increase plastic degradation [71]. For example, Jia *et al.* generated a hydrophobic cell surface display (HCSD) system in *E. coli* BL21(DE3). The HCSD system comprised a truncated FadL transmembrane protein as the membrane anchor, *ls*PETase, and a class II type hydrophobin from *Trichoderma reesei* (*Tr*HFBII) connected by flexible linkers (Fig. 2B). Surface contact angle measurements showed that PET film treated with the HCSD system had decreased surface hydrophobicity compared to samples treated with cells displaying PETase or *Tr*HFBII alone. As the hydrophobicity of PET decreases during hydrolysis, this was indicative that the co-display of *Tr*HFBII and PETase increased the PET degradation rate [56, 72]. Scanning electron microscopy (SEM) further confirmed increased PET surface modification by HCSD than PETase displayed alone. In a 7-day incubation with new media or free PETase supplemented after 92 h, the HCSD system released 52% more terephthalic acid (TA) from a sample of a commercial PET bottle compared to the free enzyme. This work showed that co-expression of hydrophobin *Tr*HFBII was not detrimental to *Is*PETase activity and further, increased plastic degradation activity through increasing cellular contact with the plastic surface.

In a separate study, Chen and co-workers engineered a cell-surface display system of *ls*PETase using GCW51 on *Pichia pastoris* GS115 yeast cells [68]. This system was enhanced by the addition of a class I hydrophobin from *Trichoderma reesei* (*Tr*HFBI)*,* which was co-displayed using the transmembrane anchor protein GCW61 to improve the adsorption of the engineered cells onto to the PET surface (Fig. 2C) [57]. The addition of *Tr*HFBI increased the hydrophobicity of the whole-cell catalyst, as shown through surface contact angle measurements and microbial adhesion to hydrocarbons (MATH) analysis. The removal of *Tr*HFBI from the co-display system decreased the rate of production of PET degradation products mono-(2-hydroxyethyl)terephthalic acid (MHET), bis(2-hydoxyethyl)terephthalate (BHET) and TA. The co-display system was active in depolymerising high crystallinity (45%) lab-grade, low crystallinity (6.3%) lab-grade and commercial PET. The co-display system exhibited approximately 10.9% degradation of high crystallinity lab-grade PET over the course of 10 days compared to only 1.2% for PETase displayed on its own.

In a complementary strategy to hydrophobins, Hu and Chen tested two hydrophobic adhesins, cp52k from *Pollicipes pollicipes* and mfp-3 from mussels. These adhesins were co-displayed on the exterior of *E. coli* BL21(DE3) cells with FAST-PETase [73] to degrade amorphous PET (Fig. 2D). The adhesins were surface-displayed through genetic fusion to a truncated ice nucleation protein (INP) and FAST-PETase was surface displayed via fusion to the autotransporter AIDA-I [58]. The sole-display of mfp-3 exhibited a 50% increase in adhesion to the plastic compared to the control. The co-display of mfp-3 with FAST-PETase exhibited 2.3-fold higher PET degradation over 24 h compared to the free FAST-PETase and 2-fold higher than sole-display of FAST-PETase at 30 °C. Whilst the efficiency of plastic degradation in these examples cannot readily be compared due to differences in the surface area: volume ratio and degree of crystallinity of the PET used, these studies show that significant increases in PET degradation rate can be achieved through co-localising the biocatalyst and plastic surface through cellular adhesion to the plastic surface.

Finally, the ability of native bacterial biofilms to degrade plastic has been widely investigated [74, 75]. In one particular example, the study of plastic-associated biofilms resulted in the discovery of novel polyester-degrading biocatalysts. In this work, Howard and McCarthy identified potential PET-degrading enzymes from modulated biofilms using homology searches with known PET-degrading enzymes [59]. Putative polyester-degrading biocatalyst candidates were screened for polycaprolactone (PCL) degradation activity, which identified two novel PCL-hydrolysing enzymes, Dh3 and Dh5. These enzymes were also discovered to degrade PET powder with > 40% crystallinity. Dh3 and Dh5 exhibited a ~ 3-fold and ~ 9-fold increase, respectively, in the production of TA and MHET from PET, compared to the control Thc_Cut2, a well-studied cutinase with PET-hydrolysis activity [59, 76]. To concentrate these PET-degrading enzymes at the PET surface, biofilm formation was upregulated via the overproduction c-di-DMP, through plasmid-based overexpression of diguanylate cyclases DgcC and WspR. Furthermore, the authors hypothesised that the biofilm EPS decreased the rate of diffusion of Dh3 and TfCut2 away from the substrate. Both Dh3 and TfCut2 exhibited 1.2-fold improved weight reduction of PCL when expressed alongside DgcC compared to the enzymes expressed singly in *E. coli.* However, testing of the induced biofilm paired with enzyme secretion for PET degradation was not reported.

## Engineered microbes for capture and degradation of microplastics

**Fig. 3:**
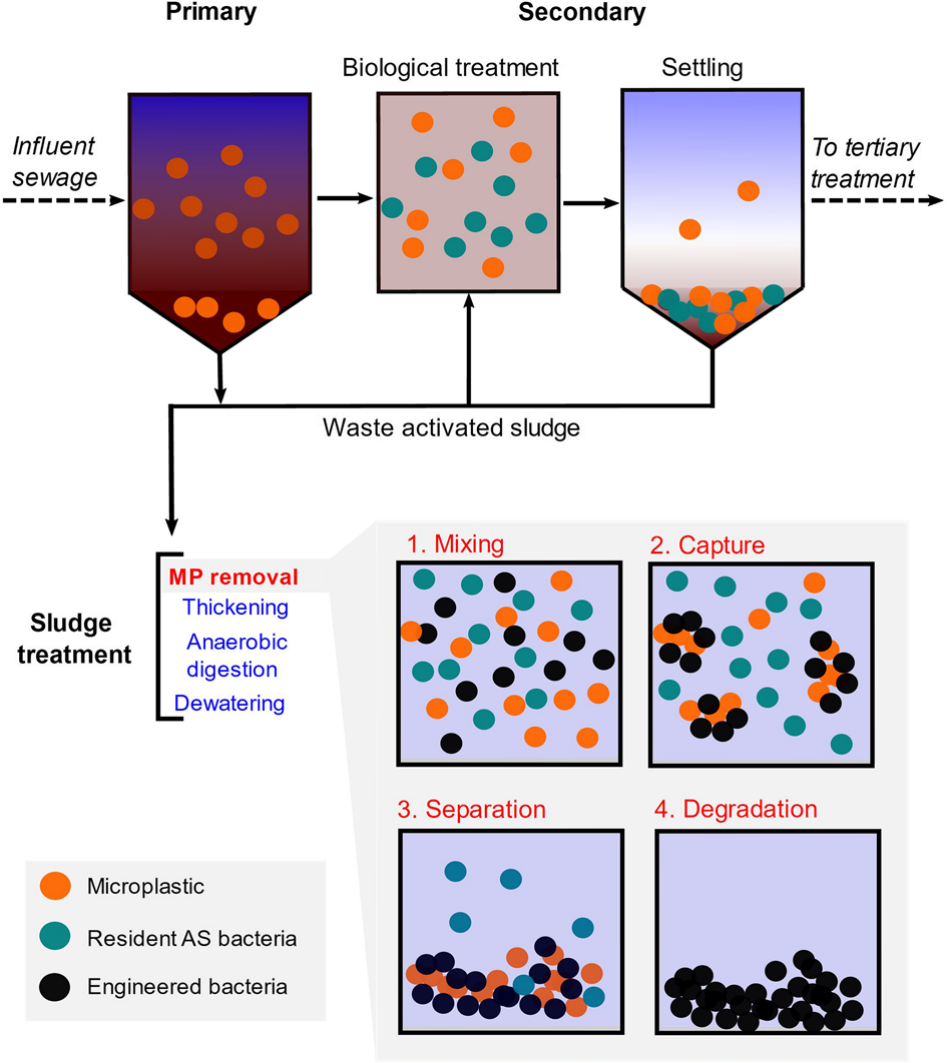
Theoretical use of engineered aggregated microorganisms for MP removal in WWT. AS: activated sludge

Whilst the examples discussed so far address microbial adhesion and degradation of macroscopic, bulk plastics, recent studies have revealed an alarming incidence of microplastics and nanoplastics (MNPs) in an incredibly diverse array of environments, including in marine organisms [77], soils [78], food [79], and even inside the human body [80, 81]. Microplastics (MPs) are defined as small pieces of plastic with a characteristic size ranging from 1 to 5000 µm, whilst nanoplastics are less than 1 µm [82]. A common source of anthropogenic release of MNPs into the environment is through municipal wastewater treatment (WWT) [83–85]. After the biological treatment step, MNPs can remain in the effluent when it is released into rivers and coastal waters (Fig. 3). Alternatively, MNPs can be captured in the sludge yielding fewer MNPs in the final effluent. This sludge is subsequently treated and then used as agricultural fertiliser, which often contains a residual level of MNPs that is distributed on the land. Therefore, there is an urgent need to develop methods to remove MNPs from wastewater during the WWT process.

A promising research direction to tackle microplastics (MPs) in aquatic environments is to use aggregated microbial communities such as biofilms. A desirable approach would be to treat the MPs in two main steps: (1) MP capture from the water using aggregating cells; then (2) release of MPs for recycling or, alternatively, further engineering the aggregated cells to effect MP degradation. Indeed, recent studies suggest that this may be possible using biotechnology. For example, Romero *et al. *used unmodified *P. aeruginosa* to capture polystyrene (PS) MPs and found that the cell-bound form of Psl, an EPS which facilitates aggregation in *P. aeruginosa* [86, 87], played an important role in cell-MP adhesion [88].

Whilst Romero and co-workers used the wild-type strain PAO1, Liu *et al.* [89] and Chan *et al.* [90] engineered the chassis organism *P. aeruginosa* to perform the capture and release steps. Both exploit the secondary signalling messenger c-di-GMP, which is strongly implicated in biofilm formation in the chassis organism [91]. Increased levels of c-di-GMP give rise to a more “aggregated” phenotype, typically associated with increased levels of EPS (e.g. Psl, Pel, and CdrA) [92, 93]. The two studies differ in that one employs a genetic engineering approach, whilst the other employs adaptive evolution.

In the first example, Liu *et al.* targeted the *wsp* chemosensory pathway that is inextricably linked to intracellular levels of c-di-GMP [89]. The authors engineered a *wsp* mutant, *ΔwspF*, that overexpresses c-di-GMP. The resulting biofilms could capture 90% of the PVC MPs in the media. Phosphodiesterases (PDEs) degrade intracellular c-di-GMP, which subsequently leads to biofilm dispersal. To engineer the release of the MPs, the authors integrated a PDE gene, under the control of an arabinose-inducible promoter, into the *ΔwspF* mutant. Therefore, upon addition of arabinose, the MPs could be recovered post-biofilm dispersal. This engineered strain was shown to capture and release MPs from seawater samples collected near a sewage outfall. In the same study, the authors also implemented a similar capture and release approach in *Pseudomonas putida*.

Rather than genetically engineering bacteria to capture and release MPs, Chan and co-workers evolved *P. aeruginosa* in the presence of PS [90]. Given that PS is toxic to bacteria, growth in the presence of PS adds an evolutionary pressure to promote mutations. After 120 generations, the authors observed the emergence of a phenotype that had a propensity to aggregate microplastics. These “microplastic aggregators” (MAGs) formed biofilms rich in matrix proteins (CdrA), and were found to capture PS, PET, and PMMA MPs with sizes 1, 5, and < 106 µm respectively. The MAGs were found to possess a mutation in the genes involved in c-di-GMP synthesis such that they expressed higher levels of c-di-GMP and produced increased amounts of the adhesion protein CdrA. Addition of the protease trypsin disrupted the biofilms and released the MPs.

These two examples highlight the potential of using engineered microbes to capture MPs from aquatic environments and then subsequently separate them from the microbial community to be recycled. This provides a promising starting point for the development of novel biotechnologies for WWT, where a number of different strategies have already been proposed to remove MPs from effluents prior to dispersal in the environment [82–84, 94]. An attractive option would be to engineer microbes to capture and treat the MPs in WWT through incorporating this novel biotechnology downstream of the secondary treatment step, such as during sludge treatment (Fig. 3). In this hypothetical process, influent sewage containing MPs passes through a typical activated sludge process with primary and secondary treatment stages. In the biological treatment step, the resident microbial population consume dissolved organics and pollutants in the mixed liquor. After flocculation and settling in the secondary clarifier, cleaner effluent moves to tertiary treatment. Settled sludge containing MPs is then processed in the sludge treatment stage. We propose that MP removal could be integrated with this sludge treatment step as follows: (1) the sludge containing resident-activated sludge (AS) microbes and MPs are mixed with the engineered bacteria under limited nutrient conditions to encourage plastic biodegradation and bio-assimilation of the plastic; (2) depending on the size of the MPs, the engineered bacteria capture the MPs by either trapping them in the EPS matrix or forming biofilms directly on the MP surface; (3) the biofilm-aggregated MPs are separated; and (4) proliferation of engineered bacteria under nutrient-limited conditions to enrich the culture in plastic-degrading strains (Fig. 3). Of course, this biotechnology step would have to be designed to eliminate risk of engineered microbes escaping the WWT plant into the environment. Therefore, these designer biofilms and their translational potential should only be explored with input and cooperation from stakeholders within the water sector [95].

## Future perspectives

The global research’s focus on discovery (e.g. bioprospecting and metagenomic database mining) and engineering (including directed evolution and rational design) is expected to deliver an increasingly powerful suite of biocatalysts to enable plastic depolymerisation under ambient conditions. Research in this field could be further accelerated by the availability of high-throughput techniques for screening or selection of candidates with high stability to industrially relevant conditions, high *k*_cat_ and recyclability. These biocatalysts could then be readily integrated into surface display or secretion methods and the microbes localised to the plastic surface using various methods, as described above. To further develop these technologies towards a useful tool for plastic bioremediation, however, we propose a number of outstanding challenges and suggested research directions. These assume prior harvesting of plastic waste from the environment via large-scale clean-up operations [96, 97], or preferably direct acquisition of plastic waste after consumer or industrial use. First, the development of low-cost, low-energy and scalable pre-treatment technologies will be critical to enabling the success of bio-based plastic degradation. This is particularly pertinent in the context of methods that depend upon the adhesion of engineered microbes to plastic surfaces, where the surface properties are critical to adhesion efficiency [37, 49, 98]. Second, the characterisation of biocatalyst longevity and regeneration within these living biofilm-type communities and the establishment of a suitable feeding regime to maximise degradation productivity and minimise process costs will further advance this technology towards application. Finally, these technologies should be developed with careful consideration to future scale-up. For example, a genetic ‘kill’ switch [99–101] could be engineered into the systems as a control measure to prevent biofouling and rapidly disperse biofilms at the end of the process. Physical pre-treatment of plastic waste, such as shredding, should also be considered for deployment of these technologies in large-scale bioreactors. Finally, we note a lack of standardisation of reporting plastic degradation efficiency across the literature. To further this field and enable rapid comparison of plastic-degrading technologies, we recommend a consistent ‘industry standard’ for reporting biodegradation efficiency.

## Conclusions and outlook

Accumulation of recalcitrant plastic in the environment is a widely acknowledged global crisis and efficient, cost-effective and sustainable new technologies are urgently required to tackle the issue. Biotechnology holds huge potential to deliver sustainable methods to upcycle plastic waste into a range of industrially valuable second-generation products. However, a requisite first step to these processes is the degradation of the polymer into small molecules which may be re-polymerised into a pseudo- ‘virgin’ polymer, or further modified into a target chemical of interest. This review has demonstrated how this process can be expedited by leveraging natural mechanisms of cellular adhesion to abiotic surfaces, such as components of biofilm formation, hydrophobins and adhesins. These have been used to co-localise plastic-degrading enzymes to plastic surfaces, as well as a method for ‘catch and release’ of microplastics. In comparison to use of purified enzymes, engineering plastic-associated bacteria as a platform for remediation of plastic waste is low cost, offers improved biocatalyst longevity and shows increased degradation efficiency owing to localisation of the biocatalyst to the plastic surface. These early examples focusing on PET degradation are a promising starting point for this nascent technology, which could now be further explored for other plastic types and in the context of WWT.

## Data Availability

No datasets were generated or analysed during the current study.
